# Structural basis of retinal membrane guanylate cyclase regulation by GCAP1 and RD3

**DOI:** 10.3389/fnmol.2022.988142

**Published:** 2022-09-08

**Authors:** James B. Ames

**Affiliations:** Department of Chemistry, University of California, Davis, Davis, CA, United States

**Keywords:** calcium, GCAP1, GCAP5, RD3 protein, guanylate cyclase (guanylyl cyclase), NMR

## Abstract

Retinal membrane guanylate cyclases (RetGC1 and RetGC2) are expressed in photoreceptor rod and cone cells, where they promote the onset of visual recovery during phototransduction. The catalytic activity of RetGCs is regulated by their binding to regulatory proteins, guanylate cyclase activating proteins (GCAP1-5) and the retinal degeneration 3 protein (RD3). RetGC1 is activated by its binding to Ca^2+^-free/Mg^2+^-bound GCAP1 at low cytosolic Ca^2+^ levels in light-activated photoreceptors. By contrast, RetGC1 is inactivated by its binding to Ca^2+^-bound GCAP1 and/or RD3 at elevated Ca^2+^ levels in dark-adapted photoreceptors. The Ca^2+^ sensitive cyclase activation helps to replenish the cytosolic cGMP levels in photoreceptors during visual recovery. Mutations in RetGC1, GCAP1 or RD3 that disable the Ca^2+^-dependent regulation of cyclase activity are genetically linked to rod/cone dystrophies and other inherited forms of blindness. Here I review the structural interaction of RetGC1 with GCAP1 and RD3. I propose a two-state concerted model in which the dimeric RetGC1 allosterically switches between active and inactive conformational states with distinct quaternary structures that are oppositely stabilized by the binding of GCAP1 and RD3. The binding of Ca^2+^-free/Mg^2+^-bound GCAP1 is proposed to activate the cyclase by stabilizing RetGC1 in an active conformation (R-state), whereas Ca^2+^-bound GCAP1 and/or RD3 inhibit the cyclase by locking RetGC1 in an inactive conformation (T-state). Exposed hydrophobic residues in GCAP1 (residues H19, Y22, M26, F73, V77, W94) are essential for cyclase activation and could be targeted by rational drug design for the possible treatment of rod/cone dystrophies.

## Introduction

**FIGURE 1:**
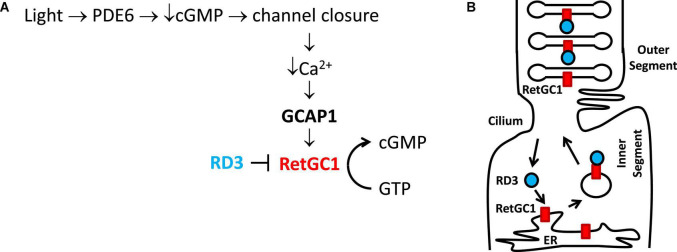
Physiological role of GCAP1 and RD3 in visual phototransduction. Adapted from Ames (2019). **(A)** Visual excitation pathway in retinal photoreceptor cells. Light-activated channel closure promotes a drop in cytosolic Ca^2+^ level that in turn causes Ca^2+^-free/Mg^2+^-bound GCAP1 to bind and activate RetGC1. RD3 binding to RetGC1 inhibits the cyclase activity. **(B)** RD3 (cyan) guides the trafficking of RetGC1 (red) to outer segment disk membranes.

Light activation of retinal rod and cone cells (called visual phototransduction) triggers a decrease in the cytosolic cGMP concentration that causes cyclic nucleotide gated (CNG) channels to close, which hyperpolarizes the plasma membrane to generate a neural signal (Stryer, 1991; Baylor, 1996; Figure 1). In dark-adapted photoreceptors, relatively high levels of cGMP keep CNG channels open, resulting in high cytosolic Ca^2+^ levels at or near 500 nM (Woodruff et al., 2002). By contrast, light-activation of the photoreceptor promotes the hydrolysis of cGMP and the light-induced lowering of cGMP causes CNG channels to close, resulting in a nearly 10-fold drop in the cytosolic Ca^2+^ level (Gray-Keller and Detwiler, 1994). The light-induced decrease in both cGMP and Ca^2+^ are important signals that promote the re-synthesis of cGMP by the enzyme retinal membrane guanylate cyclases (Dizhoor et al., 1994; Lowe et al., 1995). The re-synthesis of cGMP by the cyclase during visual recovery is highly regulated by the light-dependent cytosolic Ca^2+^ concentration (Koch and Stryer, 1988; Koutalos and Yau, 1996), and the Ca^2+^-dependent regulation of the cyclase is mediated by guanylate cyclase activating proteins discussed below. Retinal membrane guanylate cyclase is also regulated by the retinal degeneration 3 (RD3) protein (Friedman et al., 2006). The binding of RD3 to the cyclase has been shown to inhibit the cyclase enzymatic activity (Peshenko et al., 2011). In addition, RD3 binding promotes trafficking of RetGC1 from the endoplasmic reticulum (ER) to the rod outer segment disk membrane (Azadi et al., 2010; Zulliger et al., 2015).

**FIGURE 2:**
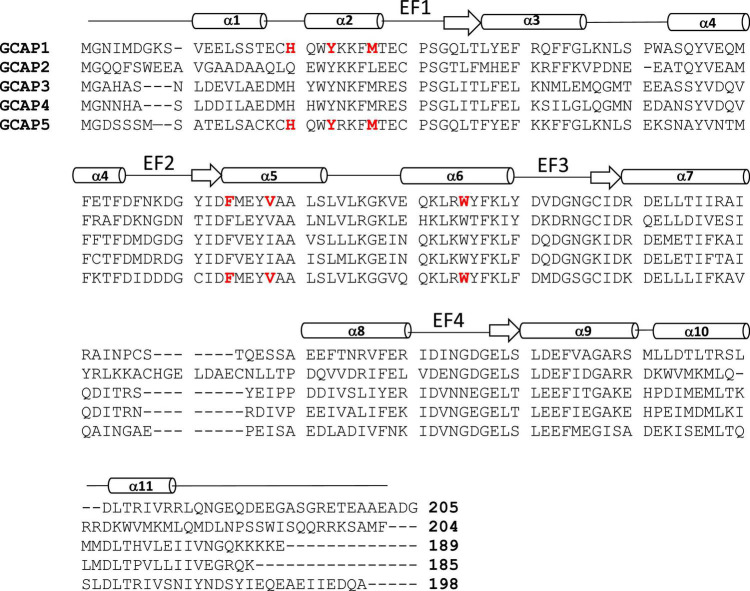
Amino acid sequence alignment of GCAP proteins. Swiss Protein Database accession numbers are P46065 (bovine GCAP1), P51177 (bovine GCAP2), O95843 (zebrafish GCAP3), Q6ZM98 (zebrafish GCAP4), and Q5MAC8 (zebrafish GCAP5). Exposed “hot spot” residues in GCAP1 and GCAP5 are highlighted red. Secondary structure (helices and strands) are shown above the sequence alignment depicted as cylinders and arrows.

Retinal membrane guanylate cyclases (RetGC1 and RetGC2) (Dizhoor et al., 1994; Palczewski et al., 1994, 2004) are regulated by a family of guanylate cyclase activating proteins (GCAP1-5, see Figure 2). RetGC1 is known to interact with GCAP1 (Laura et al., 1996), whereas RetGC2 can interact with both GCAP1 and GCAP2 (Laura and Hurley, 1998). The functional differences between RetGC1 and RetGC2 are currently not well understood. RetGC1 knockout mice cause a loss of cone function but do not exhibit rod degeneration. By contrast, RetGC2 is important for rod function but the RetGC2 knockout has no effect on cones (Yang et al., 1999; Baehr et al., 2007; Boye et al., 2011, 2013). A related membrane guanylate cyclase (regulated by GCAP1) is also expressed in the olfactory bulb (Duda et al., 2001). RetGCs are regulated by up to 8 different vertebrate homologs of guanylate cyclase activating proteins (GCAP1-8) (Imanishi et al., 2004; Scholten and Koch, 2011). The binding of Ca^2+^-free GCAP1 to RetGC1 activates the cyclase enzymatic activity at low Ca^2+^ levels in light activated photoreceptors (Peshenko and Dizhoor, 2006; Lim et al., 2009). By contrast, the binding of Ca^2+^-bound GCAP1 to RetGC1 inhibits the cyclase activity at high Ca^2+^ levels in dark-adapted photoreceptors (Dizhoor and Hurley, 1996; Dizhoor et al., 1998). The Ca^2+^-bound GCAP1 can also activate the odorant surface receptor ONE-GC (Duda et al., 2012), which raises the question about how Ca^2+^-bound GCAP1 can oppositely regulate both RetGC1 and ONE-GC. The Ca^2+^-dependent regulation of RetGCs by GCAPs in the retina coordinates the recovery phase of visual phototransduction (Figure 1A). Light-activation of the photoreceptor causes a reduction in both cGMP and Ca^2+^ levels (Figure 1A), which triggers a need for RetGC1 to become activated by Ca^2+^-free GCAP1 when the cytosolic Ca^2+^ and cGMP levels are both low. This light-induced activation of RetGC1 is important for replenishing cGMP levels to recover the dark state during visual recovery (Figure 1A). The Ca^2+^-dependent cyclase regulation is abolished in GCAP1 knockout mice (Burns et al., 2002; Howes et al., 2002; Mendez and Chen, 2002; Pennesi et al., 2003). Particular GCAP1 mutants that weaken Ca^2+^ binding to GCAP1 (Dell’orco et al., 2010) cause constitutive activation of RetGC1 that directly leads to retinal degenerative diseases known as rod-cone dystrophies (Semple-Rowland et al., 1996; Sokal et al., 1998; Baehr and Palczewski, 2007; Behnen et al., 2010; Bondarenko et al., 2010; Jiang and Baehr, 2010; Dell’orco et al., 2014).

RD3 is a 23-kDa retinal protein that is essential for proper photoreceptor function, and the deletion of RD3 causes retinal degeneration and blindness in human patients that possess recessive Leber Congenital Amaurosis 12 (LCA) (Friedman et al., 2006). The lack of RD3 expression in knockout mice leads to reduced levels of RetGC1 in the outer segment membrane of photoreceptors. This reduction of RetGC1 caused by a lack of RD3 is consistent with observations that RD3 promotes the trafficking of RetGC1 into the outer segment (Azadi et al., 2010; Zulliger et al., 2015; Figure 1B). The RetGC1 protein is first expressed inside the endoplasmic reticulum (ER), where it is processed and inserted into transport vesicles (Figure 1B). The RetGC1 containing transport vesicles in the inner segment then bind to RD3, which guides the trafficking of these vesicles into the outer segment. The binding of RD3 to RetGC1 facilitates the transfer of RetGC1 from transport vesicles into the outer segment disk membrane. RD3 binding to RetGC1 also inhibits the cyclase enzymatic activity, perhaps by obstructing the binding of GCAPs (Peshenko et al., 2011, 2016; Figure 1A). The RD3 binding site in RetGC1 involves multivalent contacts. Mutagenesis studies have suggested that RD3 contacts the RetGC1 dimerization domain (residues 800–851) that also interacts with GCAP1 (Peshenko et al., 2011, 2016). RD3 also interacts with a C-terminal region in RetGC1 that is downstream of the catalytic domain (Azadi et al., 2010). The RD3 binding to RetGC1 is important for keeping the cyclase enzymatic activity turned off during its vesicle trafficking to the disk membrane. The RetGC1 trafficking in the absence of RD3 causes elevated cGMP levels in the inner segment, which turns on apoptosis that leads to retinal degeneration (Dizhoor et al., 1998; Newbold et al., 2002). As a result, mutations in RD3 and/or RetGC1 that disable RD3 binding are believed to cause Lebering are belie amaurosis (Azadi et al., 2010; Zulliger et al., 2015) and various forms of retinal degeneration (Friedman et al., 2006; Azadi et al., 2010; Molday et al., 2013, 2014). The ability of RD3 to prevent aberrant activation of RetGC1 by GCAPs is essential for the survival of photoreceptors (Peshenko et al., 2016). Lastly, RD3 may also interact with guanylate kinase to promote the synthesis of GDP to control the recycling of nucleotides in the inner segment (Wimberg et al., 2018). However, more recent studies argue against any role for RD3 in GMP recycling (Dizhoor et al., 2021).

In this review, I provide an overview of previous atomic-resolution structures of GCAP1 (Stephen et al., 2007; Lim et al., 2016), GCAP5 (Cudia et al., 2021) and RD3 (Peshenko et al., 2019), and propose a molecular mechanism for how RetGC1 might be regulated by GCAP1 and RD3.

## Molecular structure and function of guanylate cyclase activating proteins

### Guanylate cyclase activating proteins are a family of retinal Ca^2+^ sensors

**FIGURE 3:**
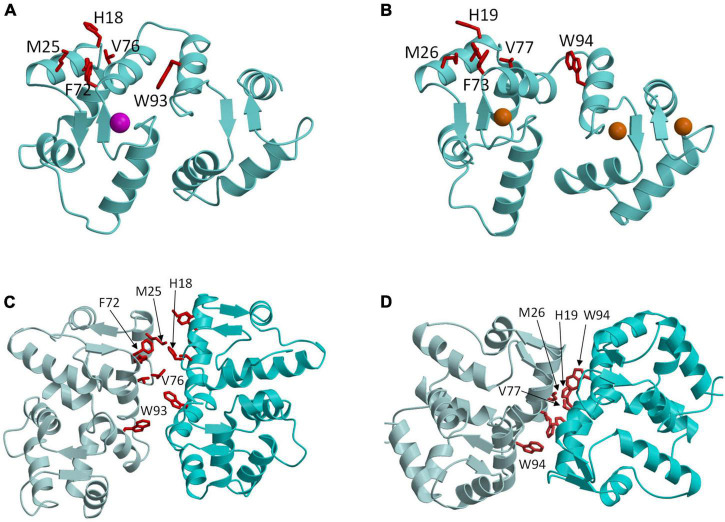
Molecular structures of GCAP1 and GCAP5. Main chain structures of Ca^2+^-free/Mg^2+^-bound GCAP5 (PDB ID: 7M2M) **(A)** and Ca^2+^-bound GCAP1 (PDB ID: 2R2I) **(B)**. Bound Ca^2+^ and Mg^2+^ are orange and magenta. Dimeric structures of GCAP5 **(C)** and GCAP1 **(D)** determined by double electron electron resonance (DEER). Side chain atoms of exposed “hot spot” residues are shown as red sticks.

The GCAP proteins (GCAP1-5, see Figure 2) are a family of Ca^2+^ binding proteins expressed exclusively in vertebrate photoreceptors (Polans et al., 1996; Ames, 2021). GCAP1 (Palczewski et al., 1994) and GCAP2 (Dizhoor et al., 1995) were originally discovered in mammalian photoreceptors, but up to seven GCAP homologs were later discovered in zebrafish and other vertebrate species (Imanishi et al., 2004; Scholten and Koch, 2011). The GCAP proteins consist of about 200 amino acid residues that contain N-terminal myristoylation, four EF-hand Ca^2+^ binding sites (EF1, EF2, EF3, and EF4 in Figure 2), and non-conserved residues in the N-terminal and C-terminal helices (α1 and α11 in Figure 2). The EF-hands in GCAP1 can bind to both Ca^2+^ and Mg^2+^ (Peshenko and Dizhoor, 2006, 2007). Mg^2+^ can bind to the second EF-hand (magenta sphere in Figure 3A) in GCAP1 (Peshenko and Dizhoor, 2004, 2007; Lim et al., 2009), which stabilizes the Ca^2+^-free/Mg^2+^-bound protein conformation (Figure 3A) that activates RetGC1 in light-activated photoreceptors (Dizhoor et al., 1994; Peshenko and Dizhoor, 2004; Marino et al., 2015). In dark-adapted photoreceptors, Ca^2+^ binds to GCAP1 (in place of Mg^2+^), which stabilizes the Ca^2+^-bound structure (Figure 3B) that is important for the inhibition of RetGC1 (Dizhoor et al., 1998). Ca^2+^ binds to GCAP1 at the second, third and fourth EF-hands (orange spheres in Figure 3B; Ames et al., 1999; Stephen et al., 2007) with an apparent dissociation constant of 100 nM (Lim et al., 2009; Dizhoor et al., 2010). Dark-adapted photoreceptors maintain relatively high cytosolic Ca^2+^ levels (500 nM) (Woodruff et al., 2002), which promotes the formation of Ca^2+^-bound GCAP1 (Dizhoor and Hurley, 1996; Dizhoor et al., 1998). Light-activation of the photoreceptor causes a 10-fold reduction in the cytosolic Ca^2+^ level (Gray-Keller and Detwiler, 1994; Woodruff et al., 2002) while the Mg^2+^ concentration remains fixed at ∼1 mM (Chen et al., 2003). Therefore, GCAP1 binds to Mg^2+^ instead of Ca^2+^ in light-activated photoreceptors, and the Ca^2+^-free/Mg^2+^-bound GCAP1 binds to RetGC1 to promote the synthesis of cGMP during visual recovery (Dizhoor et al., 1994, 1995; Gorczyca et al., 1995).

### GCAP1 and GCAP5 form dimers in the absence of retinal membrane guanylate cyclases 1

GCAP1 and GCAP5 both form homodimers in solution in the absence of RetGC1 (Figures 3C,D). The dimerization of GCAP1 and GCAP5 are both Ca^2+^-independent and have a dimerization dissociation constant in the micromolar range (Lim et al., 2018; Boni et al., 2020; Cudia et al., 2021). Structural models of GCAP1 and GCAP5 dimers were both determined previously by measuring intermolecular DEER distances that served as restraints for molecular docking (Lim et al., 2018; Cudia et al., 2021; Figures 3C,D). The overall structures of both dimers are fairly similar (RMSD = 2.4 Å) and residues at the dimer interface are highly conserved (see red residues in Figure 3). An important structural difference is that the GCAP5 dimer forms an intermolecular salt bridge between R22 and D71 that is not seen in the GCAP1 dimer, and the GCAP5 mutation R22A abolishes dimerization (Cudia et al., 2021). The dimer structures of GCAP1 and GCAP5 are both stabilized by hydrophobic contacts at the dimer interface (Figures 3C,D). The most prominent intermolecular contacts involve conserved hydrophobic residues, H19, Y22, M26, F73, V77, and W94 in GCAP1 (Figure 3). In particular, the methyl side-chain atoms of V77 (V76 in GCAP5) each contact one another at the dimer interface and therefore explain why the V77E (or V76E in GCAP5) mutation significantly weakens protein dimerization for both GCAP1 and GCAP5 (Lim et al., 2016; Cudia et al., 2021). Individual point mutations at the dimer interface in GCAP1 (H19A, Y22A, F73A, V77E, and W94A) or GCAP5 (H18A, Y21A, F72A, V76E, and W93E) each weaken the dimerization dissociation constant and completely abolish the activation of RetGC1 by GCAP1 (Lim et al., 2018) or GCAP5 (Cudia et al., 2021). Thus, the exposed hydrophobic residues at the dimer interface are essential for both GCAP dimerization and activation of RetGC1.

### Mutational hotspot residues in GCAP1 and GCAP5

**FIGURE 4:**
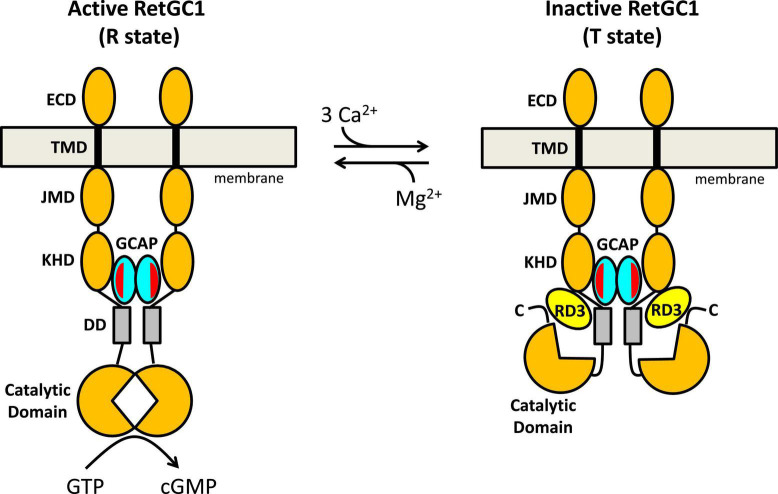
Schematic model of RetGC1 (orange) regulation by GCAP1 (cyan) and RD3 (yellow). Abbreviations of RetGC1 domains: ECD, extracellular domain; TMD, transmembrane domain; JMD, juxtamembrane domain; KHD, kinase homology domain; DD, dimerization domain (gray). The exposed hydrophobic patch in GCAP1 is red.

The structures of GCAP1 (Figure 3B; Stephen et al., 2007) and GCAP5 (Figure 3A; Cudia et al., 2021) reveal exposed hydrophobic residues (H19, Y22, M26, F73, V77, and W94 in GCAP1 or H18, Y21, F72, V76, and W93 in GCAP5) that I propose could be targeted by rational drug design (see exposed residues highlighted red in Figure 3). The exposed hydrophobic residues are located at the dimer interface for both GCAP1 and GCAP5 (Figures 3C,D). Mutating the exposed residues in GCAP1 (H19A, Y22A, M26A, F73A, V77E, and W94E) each weaken dimerization and abolish cyclase activation (Lim et al., 2018). The corresponding mutations in GCAP5 (H18E, Y21E, M25E, F72E, V76E, and W93E) also weaken dimerization and abolish cyclase activation (Cudia et al., 2021). These results initially suggested that the dimeric structures of GCAP1 (and GCAP5) might be essential for cyclase activation. However, the GCAP5 mutant (R22A) was recently shown to abolish GCAP5 dimerization but still caused a threefold activation of the cyclase, which suggests that formation of a GCAP5 dimer (as seen in Figure 3C) may not be required to activate the cyclase. The enhanced cyclase activation caused by the monomeric R22A mutant is consistent with a previous suggestion that the exposed hotspot residues in GCAP1 may interact directly with RetGC1 (Peshenko et al., 2014) rather than mediate GCAP dimerization. If the exposed hotspot were to bind to RetGC1 with higher affinity than it binds to itself, then the apparent dimerization of GCAP1 and GCAP5 that occurs in the absence of RetGC1 might be an artifact of not having the cyclase present. Future studies are needed to test whether RetGC1 binding to GCAP1 prevents GCAP1 dimerization to distinguish whether the GCAP1 hotspot binds to RetGC1. Regardless of whether the exposed hotspot facilitates GCAP1/GCAP5 dimerization (Figure 3) or binds to RetGC1 (Figure 4), this hotspot (highlighted red in Figure 4) could serve as a binding site for an inhibitor whose binding would increase the apparent K_*m*_ value of the cyclase activity. The binding of small molecules or peptides that selectively target the hotspot should prevent GCAP1 from activating RetGC1. In particular, small molecule inhibitors that selectively target the hot spot of constitutively active GCAP1 mutants [Y99C (Payne et al., 1998), D100G (Nong et al., 2014), E111V (Marino et al., 2018), and E155G (Wilkie et al., 2001)] should block the constitutive activation of RetGC1. Future studies are needed to screen for small molecule inhibitors that specifically target the exposed hotspot in GCAP1 mutants and test whether these drugs might slow the progression of cone-rod dystrophies.

## Structural basis of rd3 binding to retinal membrane guanylate cyclases 1

### Retinal degeneration 3 forms an elongated four-helix bundle

**FIGURE 5:**
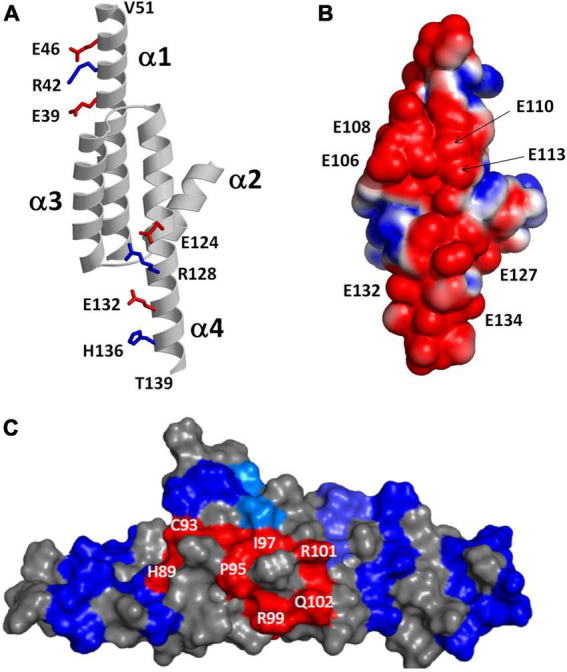
Structural analysis of RD3. Adapted from Peshenko et al. (2019). **(A)** Main chain structure of RD3. Salt bridges near the N-terminus (E39, R42, and E46) and C-terminus (E124, R128, E132, and H136) rigidify α1 and α4 to form an elongated 4-helix bundle. **(B)** Map of surface electrostatic potential of RD3 illustrates negatively charged protein surface (red). **(C)** Space-filling representation of RD3 shows residues on the protein surface (highlighted in red) that contact RetGC1.

The NMR structure of RD3 (Peshenko et al., 2019) reveals an elongated overall structure (70 Å long by 30 Å wide) that adopts a four-helix bundle (helix α1: P21–V51; α2: P75–K87; α3: P90–Q107; α4: V111–T139) (Figure 5A). The four helices in RD3 are bundled together in an antiparallel fashion with interhelical contacts formed by hydrophobic residues on the inner surface of helices α1 (residues L29 and L33), α3 (F100), and α4 (V114, F118, L122) whose side chain atoms point inward toward the hydrophobic core. The N-terminal and C-terminal ends of helices α1 and α4 are solvent exposed and the ends of these helices are rigidified by a series of salt bridge interactions (see blue and red side chain atoms in Figure 5A), which generates a long end-to-end distance in the elongated structure. A map of the electrostatic surface potential of RD3 reveals a negatively charged protein surface on one side of the protein (Figure 5B), in which many negatively charged glutamate side chain atoms (from E106, E108, E110, E113, E127, E132, E134) are clustered together on the protein surface and are suggested to make electrostatic contacts with RetGC1. A separate set of exposed residues in RD3 (see H89, C93, P95, I97, R99, R101, Q102 in Figure 5C) are clustered on the opposite side of the protein surface located near the center of the structure (highlighted red in Figure 5C). Site-specific mutations of these solvent exposed residues (see H89, C93, P95, I97, R99, R101, Q102, S120 in Figure 5C) have been shown to abolish RetGC1 regulation (Peshenko et al., 2016), suggesting that these residues might make direct contact with RetGC1.

### Retinal degeneration 3 binding to retinal membrane guanylate cyclases 1

The three-dimensional structure of RD3 (Figure 5) provides clues about how RD3 might bind to and regulate RetGC1. Previous studies suggested that RD3 and GCAP1 may competitively bind to RetGC1 (Peshenko et al., 2011, 2016). The RD3 binding site on RetGC1 was therefore initially suggested to overlap at least partially with the GCAP1 binding site in RetGC1. GCAP1 and GCAP2 have been shown to interact primarily with the kinase homology and dimerization domains within RetGC1 (Peshenko et al., 2015a,b). The W708R mutation within the kinase homology domain of RetGC1 was recently shown to abolish RetGC1 binding with both RD3 and GCAP1, which is consistent with a partial overlapping of the GCAP1 and RD3 binding sites (Peshenko et al., 2015b). However, RD3 binding to RetGC1 was also disrupted by the removal of a C-terminal fragment downstream of the cyclase catalytic domain (Azadi et al., 2010). This same C-terminal deletion does not affect RetGC1 binding to GCAP1 (Peshenko et al., 2015b). Furthermore, particular point mutations in the RetGC1 dimerization domain (R822A and M823A) that block RetGC1 binding to GCAP1 have no effect on RetGC1 binding to RD3 (Peshenko et al., 2015a). These different binding properties suggest that RD3 and GCAP1 may have non-overlapping binding regions or perhaps a non-localized and multivalent binding site in each case. Future studies are needed to map the entire RD3 binding site in RetGC1.

## Allosteric mechanism of retinal membrane guanylate cyclases 1 regulation by guanylate cyclase activating proteins 1 and retinal degeneration 3

A schematic model for the allosteric regulation of RetGC1 by GCAP1 and RD3 is presented in Figure 4. RetGC1 is known to form a dimer in the disk membrane (Liu et al., 1997; Olshevskaya et al., 1999; Yu et al., 1999). The dimeric RetGC1 is proposed to adopt two distinct quaternary structures: The R-state conformation places the two catalytic domains in close proximity to assemble the cyclase active site at the dimer interface in order to provide maximal enzymatic activity (Figure 4, left panel). The T-state conformation causes the catalytic domains to disassociate and disassemble the cyclase catalytic site, which should abolish the cyclase activity (Figure 4, right panel). The dimeric RetGC1 is believed to bind two molecules of GCAP1 to form a 2:2 complex (Peshenko et al., 2010). Previous studies have observed that GCAP1 may adopt a pre-formed dimer before binding to the RetGC1 dimer (Lim et al., 2018; Boni et al., 2020). However, more recent studies suggest that GCAP5 dimerization is not required for cyclase binding and activation (Cudia et al., 2021). Therefore, the exposed hydrophobic patch in GCAP1 (red residues in Figure 3) that facilitates GCAP1 dimerization in the absence of RetGC1 (Figures 3C,D) is proposed here to interact directly with RetGC1 as suggested previously (Peshenko et al., 2014) (see red patch in Figure 4). RetGC1 binding to Ca^2+^-free/Mg^2+^-bound GCAP1 at low Ca^2+^ levels (in light-activated photoreceptors) is proposed to stabilize the RetGC1 dimer in the active conformation (R), which shifts the equilibrium in favor of the active R state and turns on cyclase activity. The binding of Ca^2+^-bound GCAP1 to RetGC1 at high Ca^2+^ levels (in dark-adapted photoreceptors) is proposed to stabilize the RetGC1 dimer in the inactive conformation (T). In essence, GCAP1 is suggested here to serve as both a positive and negative allosteric effector for RetGC1: Ca^2+^-free GCAP1 shifts the allosteric equilibrium in favor of R, in contrast to Ca^2+^-bound GCAP1 that shifts the equilibrium in favor of T. In the absence of GCAP1, the RetGC1 dimer can occupy both conformational states (T and R). However, the conformational equilibrium in the absence of GCAP1 is shifted toward the inactive T-state, because the basal cyclase activity (in the absence of GCAP1) is more than 100-fold lower than it is in the presence of GCAP1.

The structural model of the RetGC1 dimer also explains how RD3 (yellow in Figure 4) might inactivate the cyclase activity (Figure 4, right panel). RD3 is proposed to bind to the RetGC1 dimer by contacting the KHD/dimerization domains (Peshenko et al., 2011, 2016) and the C-terminal region downstream of the catalytic domain (Azadi et al., 2010). This multivalent interaction is proposed to lock the RetGC1 dimer in the inactive T-state conformation. The binding of RD3 to RetGC1 (without the binding of GCAP1) must be sufficient to stabilize the inactive T-state, because RD3 alone can bind to and inactivate RetGC1 in the inner segment, which lacks GCAP1. Therefore, the binding of RD3 to inactive T-state is suggest here to be independent of the binding of Ca2 + -bound GCAP1, which would imply that the binding of either RD3 or Ca^2+^-bound GCAP1 are each sufficient to stabilize the inactive T-state. This hypothesis could be further tested by overexpressing RD3 (Peshenko et al., 2021) in the GCAP-/- double knockout strain (Pennesi et al., 2003) to verify whether the RD3 overexpression will cause lower basal RetGC1 activity and reduced dark current. Lastly, at low cytosolic Ca^2+^ levels, the binding of Ca^2+^-free/Mg^2+^-bound GCAP1 to the RetGC1 R-state (Figure 4 left panel) should prevent RD3 from binding to RetGC1, because the structural model in Figure 4 predicts that RD3 would not be able to form multivalent contacts with the active R-state. Therefore, an important prediction of this model is that Ca^2+^-free/Mg^2+^-bound GCAP1 should weaken RD3 binding to RetGC1 at low Ca^2+^ levels, in contrast to Ca^2+^-bound GCAP1 that can bind simultaneously with RD3 to the inactive T-state of RetGC1. Future studies are needed to test the predictions of the model and to determine atomic-resolution structures of RetGC1 bound to GCAP1 and RD3.

## Conclusion

Retinal membrane guanylate cyclases (RetGC1 and RetGC2) are expressed in photoreceptor rod and cone cells where they promote the visual recovery phase of phototransduction. The cyclase enzymatic activity is oppositely regulated by GCAPs and RD3. Mutations in RetGC1, GCAP1 and RD3 that disable the Ca^2+^-dependent cyclase regulation are genetically linked to retinal degenerative diseases and inherited forms of blindness. A molecular model (Figure 4) was presented that explains how a RetGC1 dimer is allosterically regulated by the binding of GCAP1 and RD3. The binding of Ca^2+^-free/Mg^2+^-bound GCAP1 is proposed to stimulate cyclase activity by stabilizing RetGC1 in an active conformational state (R), whereas RD3 binding is proposed to decrease cyclase activity by locking RetGC1 in the inactive conformational state (T). The model predicts that Ca^2+^-free GCAP1 should inhibit RD3 binding to RetGC1, in contrast to Ca^2+^-bound GCAP1 that should enhance RetGC1 binding to RD3. These predictions could be tested by overexpressing RD3 in a GCAP-/- double knockout photoreceptor and measuring its effect on dark current. Exposed hydrophobic residues in GCAP1 (residues H19, Y22, M26, F73, V77, W94) are essential for cyclase activation and could be targeted by rational drug design for the possible treatment of rod/cone dystrophies.

## Author contributions

JA wrote and conceived the entire manuscript.
